# Mitochondrial genomes of two *Polydora* (Spionidae) species provide further evidence that mitochondrial architecture in the Sedentaria (Annelida) is not conserved

**DOI:** 10.1038/s41598-021-92994-3

**Published:** 2021-06-30

**Authors:** Lingtong Ye, Tuo Yao, Jie Lu, Jingzhe Jiang, Changming Bai

**Affiliations:** 1 Key Laboratory of Aquatic Product Processing, Key Laboratory of South China Sea Fishery Resources Exploitation and Utilization, Ministry of Agriculture and Rural Affairs, South China Sea Fisheries Research Institute, Chinese Academy of Fishery Sciences, Guangzhou, 510300 China; 2 Key Laboratory of Maricultural Organism Disease Control, Ministry of Agriculture and Rural Affairs, Qingdao Key Laboratory of Mariculture Epidemiology and Biosecurity, Yellow Sea Fisheries Research Institute, Chinese Academy of Fishery Sciences, Qingdao, 266071 China

**Keywords:** Evolutionary genetics, Marine biology, Zoology

## Abstract

Contrary to the early evidence, which indicated that the mitochondrial architecture in one of the two major annelida clades, Sedentaria, is relatively conserved, a handful of relatively recent studies found evidence that some species exhibit elevated rates of mitochondrial architecture evolution. We sequenced complete mitogenomes belonging to two congeneric shell-boring Spionidae species that cause considerable economic losses in the commercial marine mollusk aquaculture: *Polydora brevipalpa* and *Polydora websteri*. The two mitogenomes exhibited very similar architecture. In comparison to other sedentarians, they exhibited some standard features, including all genes encoded on the same strand, uncommon but not unique duplicated *trnM* gene, as well as a number of unique features. Their comparatively large size (17,673 bp) can be attributed to four non-coding regions larger than 500 bp. We identified an unusually large (putative) overlap of 14 bases between *nad2* and *cox1* genes in both species. Importantly, the two species exhibited completely rearranged gene orders in comparison to all other available mitogenomes. Along with Serpulidae and Sabellidae, *Polydora* is the third identified sedentarian lineage that exhibits disproportionally elevated rates of mitogenomic architecture rearrangements. Selection analyses indicate that these three lineages also exhibited relaxed purifying selection pressures.

## Introduction

Metazoan mitochondrial genomes (mitogenomes) usually encode the set of 37 genes, comprising 2 rRNAs, 22 tRNAs, and 13 proteins, encoded on both genomic strands. Mitogenomic gene rearrangements can affect genome replication and transcription mechanisms, and produce disruptions in the gene expression co-regulation, so they should be strongly selected against^1^. Indeed, mitogenomic architecture is generally highly conserved^2^, but some unrelated lineages exhibit exponentially accelerated mitochondrial architecture rearrangement rates^2–8^.

The phylogeny of the phylum Annelida remains debated, but the latest review of its phylogeny proposed that the phylum is split into two major groups: Pleistoannelida, comprised of the subclasses Errantia and Sedentaria, and six ‘basal’ (early-branching) lineages: Sipuncula, Amphinomida, Chaetopteridae, Magelonidae, Oweniidae and *Lobatocerebrum*^9^. Unlike in other lophotrochozoan groups, the mitochondrial gene order (GO) in annelids long appeared to be relatively conserved, with an additional feature of all genes encoded on a single strand^10^. However, a more complex picture emerged during the last ten years. The early-branching lineages exhibit relatively high rearrangement rates^11^. Among the Errantia, species from the family Syllidae, several genera from the family Polynoidae, and genus *Ophryotrocha* exhibit relatively rapidly evolving mitochondrial architecture^12–14^. Among the Sedentaria, *Urechis* species exhibit somewhat rearranged GOs^15^, and species from families Sabellidae and Serpulidae exhibit highly rearranged GOs^16–18^.

The sedentarian family Spionidae (order Spionida) is one of the largest and most common polychaete families, whose members occur in a wide variety of benthic habitats. There is only one mitogenome belonging to this family available in the GenBank: *Marenzelleria neglecta*^19^. Moreover, this is the only mitogenome available in the GenBank for the entire nominal sedentarian order Spionida (the status of this order remains debated^9^). To address this dearth of data, and thereby improve our understanding of the dynamics of mitochondrial evolution in Sedentaria, we sequenced and characterised the entire mitogenomes of two congeneric Spionidae species: *Polydora brevipalpa and Polydora websteri*.

The spionid genus *Polydora* includes species that inhabit clastic sediments, shale rock, corraline algae, living coral, sponges, and mollusk shells. While most of the shell-boring polydorids do not cause harm to the host, a handful of species can cause considerable harm^20^*. Polydora websteri* Hartman in Loosanoff and Engle, 1943 is a highly invasive shell-boring species native to the Asian Pacific that nowadays occurs around the globe, mostly as a result of global trade of commercial oyster species^21^. It causes distress to the host, reduces their growth rates, makes them susceptible to parasites or diseases, and their presence (so-called ‘mud blisters’) lowers the market value of bivalves^20^. In this way, *P. websteri* causes considerable economic losses in commercial marine mollusk aquaculture globally^21,22^. The congeneric and morphologically similar *Polydora brevipalpa* Zachs, 1933 is a relatively poorly studied species with an apparently broad range throughout the North Pacific^20^. It primarily infests scallops (Pectinidae), and there is some evidence that it can also cause economic losses^22,23^. Therefore, apart from contributing to the understanding of evolutionary dynamics of the mitochondrial genome in Sedentaria, the sequencing of these two mitogenomes will also contribute useful data for future biogeographic and evolutionary studies of these two economically important polychaete species.

## Results and discussion

### Identity and phylogeny

Morphological identification was successfully corroborated using DNA barcoding: *P. brevipalpa* and *P. websteri* barcode sequences (*cox1* fragment) exhibited a similarity of 99.81% and 100% to the top conspecific barcode matches respectively. Several studies found that mitochondrial data produce artefactual relationships in annelids^9,11^, so we conducted only orientational phylogenetic analyses. As compositional heterogeneity in mitochondrial data can produce artefactual relationships in phylogenetic reconstruction^24–26^, first we tested the dataset for compositional homogeneity. All sequences included in the analysis failed the composition Chi-Square test (p-value < 5%; df = 3). Phylogenetic inference highly unorthodox relationships, with paraphyletic, Spionidae, Hirudinidae and Erpobdellidae (Supplementary File S1). Spionidae (represented by the two *Polydora* species and *M. neglecta*) were paraphyletic due to the unorthodox position of *M. neglecta* at the base of the Sedentaria. Sabellidae, Spionidae, and Serpulidae formed a clade, which is in disagreement with the accepted relationships of these families^9^.

### Mitochondrial architecture

**Figure 1:**
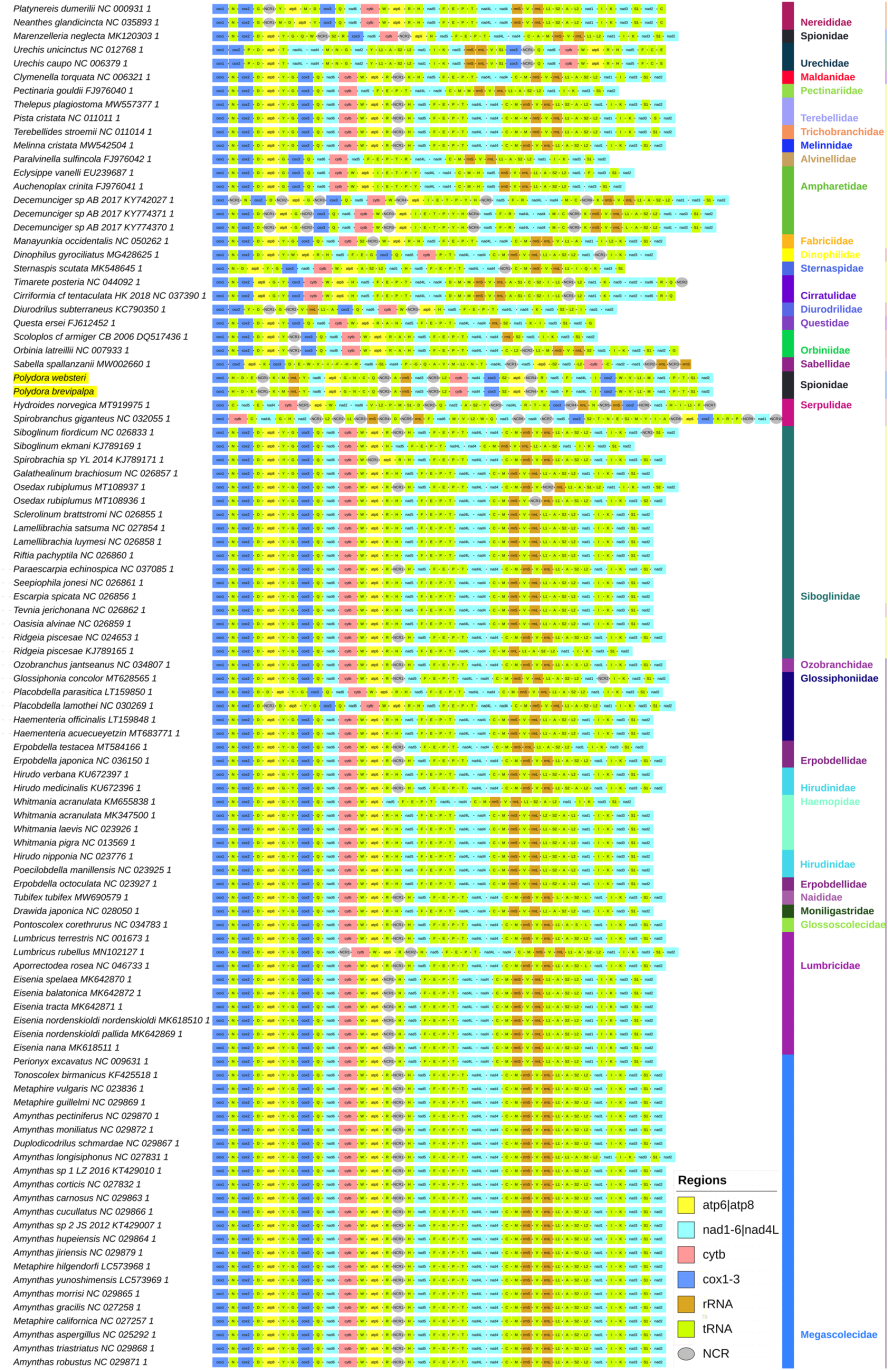
Mitochondrial architecture in the studied annelid dataset. GenBank numbers of sequences are shown next to species' names. The two newly sequenced *Polydora* species are highlighted by the yellow background. Taxonomic identity is shown to the right at the family level. The colour legend for mitogenomic architecture is shown in the figure.

The mitogenomes of *P. websteri* and *P. brevipalpa* exhibited very similar architecture and identical sizes of 17,673 bp (Table 1). This is somewhat larger than common in sedentarians, which on average have mitogenomes of around 15 Kbp (Supplementary File S2: sheet A). Only 5 sedentarian mitogenomes were larger: *Spirobranchus giganteus*^16^, *Siboglinium fiordicum*^29^ and three *Decemunciger* sp. mitogenomes^30^ . Both *Polydora* species possess the standard set of 37 genes, plus a duplicated *trnM* gene copy between *trnL1* and *nad1* (Fig. 1). Duplicated *trnM* genes have been observed in a handful of annelid species before^11,31^. The two *Polydora* aside, six more species in our sedentarian dataset exhibited a duplicated *trnM* gene (as well as some species from the early-branching lineages) (Fig. 1). However, aside from the two *Polydora* species and *S. giganteus*, in all other species the two copies were adjacent (or very near each other). As common in annelids, all genes are encoded on the same strand. Palaeoannelida, *Magelona mirabilis* (Magelonidae), and *S. spallanzanii* (Sabellida) are the only annelids described so far with genes transcribed on both strands of the mitochondrial genome^11,17^. In terms of base composition, the two *Polydora* species (AT bias of 65–66%) are average among the Sedentaria (55–78%) (Supplementary File S2: sheet A).

**Table 1:** Comparative table of the architecture of mitogenomes of *P. brevipalpa* (left) versus *P. websteri* (right).

Gene	Position		Size	IGN	Codons		Identity
	From	To			Start	Stop	%
*trnK*	1/1	66/66	66/66				93.94
*trnM*	65/65	128/128	64/64	− 2/− 2			92.19
*rrnL*	129/129	1336/1341	1208/1213				83.63
*trnY*	1337/1342	1401/1406	65/65				90.77
*nad6*	1407/1412	1895/1900	489/489	5/5	ATG/ATG	TAA/TAA	75.05
*atp8*	1898/1902	2075/2079	178/178	2/1	ATG/ATG	T–/T–	75.84
*trnG*	2071/2074	2132/2138	62/65	− 5/− 6			89.23
*trnN*	2135/2139	2202/2206	68/68	2/−			85.51
*trnC*	2202/2206	2260/2264	59/59	− 1/− 1			93.22
*trnQ*	2260/2264	2324/2328	65/65	− 1/− 1			93.85
NCR_1	2325/2329	3383/3389	1059/1061				62.48
*trnA*	3384/3390	3448/3454	65/65				83.08
*rrnS*	3449/3455	4256/4260	808/806				87.79
*nad3*	4257/4261	4616/4620	360/360		ATG/ATG	TAA/TAA	75
NCR_2	4617/4621	5275/5281	659/661				53.51
*trnL2*	5276/5282	5339/5345	64/64				84.38
*cytb*	5338/5344	6495/6501	1158/1158	− 2/− 2	ATC/ATC	TAA/TAA	80.4
*nad4*	6495/6501	7850/7856	1356/1356	− 1/− 1	ATC/ATG	TAG/TAA	74.26
*cox3*	7912/7915	8698/8701	787/787	61/58	ATA/ATC	T–/T–	80.94
*trnS2*	8775/8775	8843/8843	69/69	76/73			86.96
*atp6*	8854/8854	9559/9559	706/706	10/10	ATG/ATG	T–/T–	77.05
NCR_3	9560/9560	10,094/10,093	535/534				66.92
*trnR*	10,095/10,094	10,155/10,156	61/63				85.71
*nad5*	10,158/10,163	11,867/11,867	1710/1705	2/6	ATT/ATG	TAA/T–	75.74
*trnF*	11,871/11,869	11,934/11,934	64/66	3/1			81.82
*nad4L*	11,943/11,944	12,227/12,231	285/288	8/9	ATG/ATG	TAA/TAG	75
*trnI*	12,226/12,227	12,290/12,290	65/64	− 2/− 5			87.69
*cox2*	12,291/12,291	13,001/13,001	711/711		ATT/ATT	TAG/TAA	84.11
*trnW*	13,004/13,003	13,068/13,068	65/66	2/1			86.36
*trnV*	13,069/13,069	13,132/13,128	64/60				79.69
*trnL1*	13,133/13,134	13,198/13,198	66/65	−/5			83.33
*trnM_2*	13,199/13,199	13,263/13,263	65/65				89.23
*nad1*	13,281/13,280	14,187/14,186	907/907	17/16	ATG/ATG	T–/T–	80.26
*trnP*	14,188/14,187	14,252/14,251	65/65				89.23
*trnT*	14,254/14,252	14,316/14,315	63/64	1/−			92.19
*trnS1*	14,317/14,316	14,382/14,381	66/66				95.45
*nad2*	14,385/14,384	15,350/15,349	966/966	2/2	ATG/ATG	TAA/TAA	75.36
*cox1*	15,337/15,336	16,881/16,880	1545/1545	− 14/− 14	ATA/ATA	TAG/TAG	82.91
*trnH*	16,915/16,914	16,979/16,977	65/64	33/33			93.85
*trnD*	16,979/16,977	17,042/17,041	64/65	− 1/− 1			93.85
*trnE*	17,043/17,042	17,101/17,100	59/59				91.67
NCR_4	17,102/17,101	17,673/17,673	572/573				70.21

IGN stands for the intergenic region, where negative values indicate overlap. Intergenic regions larger than 150 bp are annotated as NCR.

The two *Polydora* species exhibited more large noncoding regions (NCRs, four were larger than 150 bp) than most other species included in the analysis (Fig. 1). This was reflected in the relatively large noncoding/coding ratio of 17.2% in their mitogenomes (Supplementary File S2: sheet A). Only *S. giganteus* and three *Decemunciger* species exhibited comparable numbers of large NCRs. NCR-1, located between *trnQ* and *trnA* was the largest with over 1000 bp, but the remaining three were also very large: 535 to 661 bp (Table 1). This is the underlying reason for the comparatively large size of these two mitogenomes. Tandem-duplication-random-loss (TDRL) events were proposed as the most likely mechanism underlying the expansion of NCRs in *S. giganteus*^16^. Given the highly rearranged architecture of these two genomes, this may also be a likely explanation for their unusually high number of large NCRs, but this remains hypothetical due to absence of evidence. We attempted to align all four large NCRs against the entire mitogenome, but none of them exhibited a similarity to the nearby coding regions. This was expected, as non-coding mitochondrial sequences tend to evolve very rapidly due to strong mutation pressures^32^, and this architecture was shared by both species, which indicates that the four NCRs appeared in the common ancestor of these two species, or even the entire genus. We also examined all 4 NCRs for open-reading-frames (ORFs). NCR1 had 5 ORFs > 50 AAs, with one large ORF spanning most of the length of the NCR (349 AAs); NCR2 had 2 ORFs > 50 AAs (57 and 58); NCR3 had only 1 ORF > 50 AAs (145); and NCR4 had 2 ORFs > 50 AAs (187 and 182). However, BLASTp analysis did not find any similarity to known proteins for any of these putative protein products, so it is unlikely that these ORFs are functional proteins.

Mitochondrial genes of Annelids exhibit a rather large variability in size^16^. Most protein-coding genes (PCGs) of the two *Polydora* species were within the range observed in other Sedentaria (Supplementary File S2: sheet B), and the two species exhibited genes of very similar size (Table 1). A few outliers in terms of gene sizes among closely related species exhibited matching insertions/deletions in both *Polydora* species, which makes it very unlikely that these were sequencing or annotation artefacts. *Cox2* was longer in these two species than in any other sedentarian mitogenome due to two insertions in the middle of the gene, and *cytb* was longer than in most other species due to an insertion at the 3’ end. The two species exhibited relatively similar start/stop codon usage (Table 1), comparable to other studied sedentarian species (Supplementary File S2: sheet B). Both species exhibited matching deletions at the 5’ end of *cox1*, which also explains the use of a different start codon (ATA) than in most other species (ATG).

On average, the most highly conserved sequences between the two genomes were exhibited by tRNA genes, only one of which (*trnV*) exhibited an identity value below 80% (Table 1). As common in mitochondrial genomes^33^, the most highly conserved PCGs were *cox* family genes, with identity values of 80–85%. Somewhat surprisingly, the *nad* gene family was the least conserved one, with identity values mostly between 74 and 76% (*nad1* was an exception: 80%). Commonly, *atp8* is the least conserved mitochondrial PCG^2,33^, but between these two species, it exhibited an identity of 75.84%, higher than most *nad* family genes. As expected, the fastest-evolving regions were NCRs, with identity values ranging from 53 to 70%.

Several genes exhibited overlaps in the two newly-sequenced mitogenomes. Genes overlapping by 1 or 2 bases have been observed in annelid mitogenomes^34^, but several overlaps in the two *Polydora* species were larger than 2 bp (Table 1). The relatively large overlap between a*tp8* and *trnG* (*P. brevipalpa* = 5 bp, *P. websteri* = 6 bp) can be explained by a 3’ end elongation of the *atp8* gene of almost 20 bases. As the 5’ end of *trnG* is relatively conserved, this suggests that the overlap arose via a mutation that affected the stop codon of the *atp8*, and caused elongation to the nearest available stop codon (T–). As *atp8* often evolves under relaxed selection constraints, it appears that this elongation did not significantly affect the fitness of the mutant phenotype. This is evidenced by both species exhibiting very similar features, which indicates that the event occurred in the common ancestor of these two species, or even the entire genus. For *nad5* in *P. websteri*, we opted for an abbreviated T– stop codon, as this produces no overlap with the adjacent *trnF* (leaves 1 bp intergenic space between the two genes). An alternative option would be to elongate the gene by 11 bases, thus creating an overlap of 10 bases with *trnF*, and use the standard TAG stop codon. The codon alignment of the shorter gene (T– stop codon) with the *P. brevipalpa* orthologue does not indicate that the gene is truncated, so we chose this as a more likely option than a large overlap. The large overlap (14 bases) between *nad2* and *cox1* putatively identified in both species is highly unusual. Usually, overlaps in metazoan mitogenomes involve tRNA genes, which is considered to be a consequence of lesser evolutionary constraints on tRNA sequences^35^. The only common overlaps between two PCGs comprise *atp6*/*atp8* and *nad4*/*nad4L*^5,34,36–40^, perhaps due to their translation from a bicistronic mRNA^34,40^. We checked DNA sequencing chromatograms for these (and all other large) overlaps and found no evidence of sequencing artefacts. An alternative option is that *nad2* uses an abbreviated stop codon T–, which would produce a 1 bp intergenic region between the two genes in *P. websteri*, and 9 bp in *P. brevipalpa*. However, abbreviated codons are usually associated with overlaps with tRNAs^41–43^, and not conserved between the two species. Given these problems, we deem the overlap to be a more likely option, but transcriptome analyses are needed to confirm this prediction.

### Gene order rearrangements

The two newly sequenced *Polydora* species exhibited completely rearranged GOs in comparison to all other available mitogenomes (Fig. 1, Table 2). Generally, GO rearrangements involving the relatively volatile tRNA genes are much more common than relatively rare PCG rearrangements^2^, but the order of PCGs was also highly rearranged in these two mitogenomes. Aside from the conserved sedentarian gene order exhibited by a majority of species (Common GO), there were 23 unique GOs in the dataset (among 97 mitogenomes). Three lineages exhibit by far the most highly rearranged GOs in comparison to the common GO: Serpulidae (represented by *S. giganteus* and *Hydroides norvegica*), *S. spallanzanii* (Sabellidae), and the two *Polydora* species. The common intervals similarity measure (where the value 1326 indicates an identical GO, and 0 indicates no shared common intervals) indicates that *S. giganteus* had the most rearranged GO (0), followed by *S. spallanzanii* (4), *H. norvegica* (6), and the two *Polydora* species (12) (Table 2). All other species exhibited much higher similarity values (≥ 90). The only other available Spionidae species, *M. neglecta*, also exhibited a unique GO, but much less rearranged than the two *Polydora* species (320). CREx analysis indicates that at least five TDRL events were necessary to explain the evolution from the common GO to the one observed in the two newly sequenced *Polydora* species (Supplementary File S3: Figure S1). The same number of TDRL events was inferred for *S. giganteus* and *H. norvegica*, but *S. spallanzanii* required a much more complex scenario (Supplementary File S3: Figures S2–S4).

**Table 2:** Gene order distances in the Sedentaria inferred using the Common Intervals measure (high numbers indicate similar gene orders).

	CGO	Ac	Cte	Cto	Dsp	Dg	Ds	Eo	Hn	Mo	Mn	Ol	Or	Ps	Pl	Psp	Qe	Ss	Ssp	Sg	Ss2	Tp	Uc	Wa
Common GO		184	94	680	186	326	246	1188	6	492	320	308	534	772	242	12	296	4	1258	0	250	1328	124	1064
*Auchenoplax crinita*	184		38	184	818	146	62	178	8	122	96	88	120	284	96	10	80	0	184	0	90	172	60	166
*Cirriformia tentaculata*	94	38		92	36	64	118	102	8	84	112	88	94	66	82	6	86	8	90	2	94	94	26	100
*Clymenella torquata*	680	184	92		184	288	162	578	6	402	226	244	380	354	172	12	252	2	614	2	196	646	82	522
*Decemunciger sp*	186	818	36	184		148	70	180	8	124	96	88	122	264	102	16	84	0	186	0	90	174	70	168
*Dinophilus gyrociliatus*	326	146	64	288	148		106	558	4	240	218	122	346	492	132	8	110	4	338	0	118	302	74	482
*Diurodrilus subterraneus*	246	62	118	162	70	106		214	6	146	116	126	246	172	214	6	124	10	246	0	88	222	114	212
*Erpobdella octoculata*	1188	178	102	578	180	558	214		6	516	274	326	834	720	378	14	302	4	1188	0	312	1256	150	1124
*Hydroides norvegica*	6	8	8	6	8	4	6	6		2	2	4	4	10	8	2	6	2	6	2	14	6	8	6
*Manayunkia occidentalis*	492	122	84	402	124	240	146	516	2		526	202	360	336	174	14	218	6	548	0	194	498	62	428
*Marenzelleria neglecta*	320	96	112	226	96	218	116	274	2	526		178	224	254	126	10	134	4	320	4	176	300	40	240
*Orbinia latreillii*	308	88	88	244	88	122	126	326	4	202	178		212	178	146	8	866	4	308	2	180	310	74	322
*Osedax rubiplumus*	534	120	94	380	122	346	246	834	4	360	224	212		504	260	8	216	6	510	0	182	496	104	768
*Paralvinella sulfincola*	772	284	66	354	264	492	172	720	10	336	254	178	504		208	12	166	0	772	0	258	720	84	628
*Placobdella lamothei*	842	184	94	502	186	404	246	1188	6	406	320	308	574	772		12	296	4	922	0	156	790	142	1064
*Polydora sp.*	12	10	6	12	16	8	6	14	2	14	10	8	8	12	8		8	14	12	6	6	12	8	16
*Questa ersei*	296	80	86	252	84	110	124	302	6	218	134	866	216	166	290	8		2	296	0	142	288	82	302
*Sabella spallanzanii*	4	0	8	2	0	4	10	4	2	6	4	4	6	0	8	14	2		4	10	2	4	8	4
*Spirobrachia sp*	1258	184	90	614	186	338	246	1188	6	548	320	308	510	772	254	12	296	4		0	214	1190	128	1064
*Spirobranchus giganteus*	0	0	2	2	0	0	0	0	2	0	4	2	0	0	0	6	0	10	0		2	0	0	0
*Sternaspis scutata*	250	86	94	196	86	114	86	312	14	194	180	180	192	268	98	6	146	2	226	2		246	46	284
*Thelepus plagiostoma*	1328	172	94	646	174	302	222	1256	6	498	300	310	496	720	264	12	288	4	1190	0	246		112	1060
*Urechis caupo*	124	60	26	82	70	74	114	150	8	62	40	74	104	84	130	8	82	8	128	0	50	112		138
*Whitmania acranulata*	1064	166	100	522	168	482	212	1124	6	428	240	322	768	628	320	16	302	4	1064	0	284	1060	138	

Column headers mirror row headers, but names are acronymic. *Polydora* sp. GO was shared by both newly sequenced *Polydora* species (*brevipalpa* and *websteri*)*. Auchenoplax crinita* is identical to *Eclysippe vanelli. Cirriformia tentaculata* is identical to *Timarete posteria.* All three *Decemucinger* sp. mitogenomes shared the same GO. *Erpobdella octoculata* is identical to *Hirudo medicinalis, Whitmania pigra, Whitmania laevis, Whitmania acranulata, Poecilobdella manillensis, Hirudo verbana* and *Hirudo nipponia. Orbinia latreillii* is identical to *Scoloplos cf. armiger*. *Paralvinella sulfincola* is identical to *Pectinaria gouldii* and *Perionyx excavatus. Placobdella lamothei* is identical to *Placobdella parasitica, Tevnia jerichonana, Siboglinum fiordicum, Siboglinum ekmani, Seepiophila jonesi, Sclerolinum brattstromi, Riftia pachyptila* and *Ridgeia piscesae. Urechis caupo* is identical to *Urechis unicinctus*. Common GO (CGO) represents the common sendentarian GO possessed by all other available mitogenomes.

If mitogenomic architecture rearrangements are strongly selected against^1^, it would be expected that elevated rearrangement rate would be associated with relaxed purifying selection pressure, which in turn should be reflected on the molecular evolution rate. To test this hypothesis, we used RELAX tool and concatenated 13 PCGs (nucleotide sequences). With all sedentarian species (and *Polydora* node) in the dataset selected as test branches (exploratory mode), the *Polydora* branch (representing the common ancestor of the two sequenced *Polydora* species) exhibited somewhat relaxed purifying selection (but not exceptional in the sedentarian dataset): k = 0.63 (where k > 1 intensified, k < 1 relaxed selection). However, the two *Polydora* species themselves exhibited highly intensified selection (k ≈ 15–17). Following this, we conducted the analysis with most species set as the reference dataset, and only the species exhibiting elevated rates of architecture rearrangements as test branches: the two *Polydora* species, *Polydora* branch, *S. spallanzanii*, *H. norvegica* and *S. giganteus*. This test for selection relaxation was significant (p = 0.00). The *Polydora* branch exhibited a highly relaxed purifying selection within the dataset (0.33), but the two *Polydora* species still exhibited intensified selection pressures (*P. websteri* k = 19.60, *P. brevipalpa* k = 18.77). The remaining three species exhibited relaxed selection pressures: *S. spallanzanii* k = 0.44, *H. norvegica* = 0.45 and *S. giganteus* k = 0.45. This corroborates that there is an association between the mitochondrial architecture rearrangement rate and purifying selection pressure in sedentarians, but the signal from *Polydora* species is rather puzzling and requires further studies.

## Conclusions

Among the Sedentaria, three lineages exhibit disproportionally highly elevated rates of mitogenomic architecture rearrangements: Serpulidae (represented by *S. giganteus*^16^ and *H. norvegica*^18^), *S. spallanzanii* (Sabellidae)^17^, and the two newly sequenced *Polydora* mitogenomes. Whereas all available Serpulidae and Sabellidae species exhibit a highly elevated mitochondrial architecture evolution rate, among the Spionidae this is limited to the genus *Polydora*. The other available species, *M. neglecta*, exhibits only a moderate gene order rearrangement rate. Intriguingly, species from these lineages formed a paraphyletic clade in phylogenetic analysis, which is most likely to be a classical example of a long-branch attraction artefact^28^. Indeed, it was previously observed that *S. giganteus* s exhibits a highly elevated evolutionary rate^16^, and proposed that this may be causing artefactual relationships in phylogenetic analyses^27^. Due to scarcity of data, the exact phylogenetic scope, and the underlying reason for, these elevated evolutionary rates remains unknown. This further supports previous observations that mitochondrial architecture is not fully conserved among the Sedentaria^16–18^, and indirectly corroborates the proposal that the evolution of mitogenomic architecture is highly discontinuous: long periods of stasis are interspersed with periods of exponentially accelerated evolutionary rate of mitogenomic rearrangements^5^. The previous observation that *S. spallanzanii* has genes encoded on both mitochondrial strands raises intriguing questions about the evolution of mitochondrial transcription mechanism in Annelida^11^, as Boore proposed a ‘ratchet’ effect that would constitute a barrier to further strand switches once the replication mechanism has been lost on one strand^40^. As introns were described in mitochondrial genes of three separate annelid lineages so far^30,44,45^, this implies that mitochondrial evolution in Sedentaria deserves more scientific attention than it is currently receiving and that further annelid mitogenomes should be sequenced in order to further elucidate the intriguing patterns of mitogenomic evolution in this class.

## Methods

### Sample, sequencing, assembly and annotation

Samples used for sequencing were procured at two different locations (Table 3). Samples were identified morphologically according to^20^ and more recent redescriptions^23,46^, as well as via *cox1* barcoding using the BOLD database^47^. As the animal handling included only unprotected invertebrates, no special permits were required to retrieve and process the samples.

**Table 3:** Sampling details.

Species	*Polydora websteri*	*Polydora brevipalpa*
Host	*Crassostrea hongkongensis*	*Mizuhopecten yessoensis*
Locality	Yangjiang, China	Dalian, China
Geographic coordinates	Long. 112.049, Lat. 21.784	Long. 122.738, Lat. 39.02
Salinity (ppt)	20	33
Habitat	Estuary	Open sea

DNA extraction, amplification, sequencing and mitogenome assembly were conducted closely following the methodology described before^6,48^. Briefly, DNA was extracted from the complete specimens using AidLab DNA extraction kit (AidLab Biotechnologies, Beijing, China). The mitogenomes were amplified and sequenced using 14 and 12 primer pairs, respectively (Supplementary File S3: Tables S1 and S2). The primers were designed to produce amplicons that overlap by approximately 100 bp. PCR reaction mixture of 50 µL comprised 5 U/µL of TaKaRa LA Taq polymerase (TaKaRa, Japan), 10 × LATaq Buffer II, 2.5 µM of dNTP mixture, 0.2–1.0 µM of each primer, and 60 ng of DNA template. PCR conditions were: initial denaturation at 98 °C for 2 min, and 40 cycles of 98 °C for 10 s, 50 °C for 15 s, and 68 °C for 1 min/kb. PCR products were sequenced using the same set of primers and Sanger method. Electropherograms were visually inspected, and amplicon identity was confirmed using BLAST^49^. The mitogenomes were assembled using DNAstar v7.1^50^, making sure that overlaps were identical and mitogenomes circular. Protein-coding genes were approximately located using DNAstar and then manually checked against the orthologous sequences using BLAST and BLASTx. tRNAs were identified using tRNAscan^51^ and ARWEN^52^ tools. The two ribosomal RNAs were manually annotated via a comparison with orthologues. The mitogenome of *Marenzelleria neglecta*^19^, the only available Spionidae representative, was used as the template for assembly and annotation. The annotation recorded in a Word (Microsoft Office) document was parsed and extracted using PhyloSuite^53^. The same program was used to generate the file for submission to GenBank.

### Dataset, comparative mitogenomic, phylogenetic, sequence and selection analyses

We used PhyloSuite^53^ to retrieve, standardize, and extract features of mitogenomes available in the GenBank; as well as standardise the annotation (gene names), semi-automatically re-annotate ambiguously annotated tRNA genes with the help of the ARWEN output, extract mitogenomic features, generate comparative tables, concatenate alignments and prepare input files for its plug-in programs. We conducted analyses on all Sedentaria (sensu^9^) mitogenomes available in the GenBank. We included two Errantia (Nereididae) species as outgroups: *Platynereis dumerilii*^54^ and *Neanthes glandicincta*^55^. A study has shown that mitochondrial data produce phylogenetic artefacts when incomplete mitogenomes are included in analysis^11^, so we removed seven mitogenomes that exhibited missing PCGs (small genes, like *atp8*, were ignored in this case). All phylogenetic analysis steps were conducted using PhyloSuite and its plug-in programs. Amino acid sequences of 13 PCGs were aligned in batches using the accurate 'G-INS-i strategy implemented in MAFFT^56^. Maximum likelihood phylogeny was inferred using IQ-TREE^57^, with 20,000 ultrafast bootstraps^58^. This program uses ModelFinder^59^ for the optimal model selection, and phylogenetic terrace aware data structure for the efficient analysis under partition models^60^. Phylograms and gene orders were visualized and annotated (using files generated by PhyloSuite) in iTOL^61^. MEGA-X^62^ was used to attempt to align NCR sequences to the rest of the mitogenomes, and ORFfinder^63^ was used to search for ORFs in the NCRs. CREX was used to infer the GO distances^64^. RELAX, available from the Datamonkey server^65^, was used to test whether the strength of natural selection has been statistically significantly relaxed or intensified along a specified set of test branches^66^. We used concatenated nucleotide sequences of 13 PCGs for this analysis.

### Ethics declaration

As the animal handling included only unprotected invertebrates, no special permits were required to retrieve and process the samples.

## Supplementary Information

Supplementary Information 1.

Supplementary Information 2.

Supplementary Information 3.

## Data Availability

All data generated or analysed during this study are included in this published article, its supplementary information files, and the NCBI’s GenBank repository under the accession numbers MW316633 (*P. websteri*) and MW316635 (*P. brevipalpa*).

## Abbreviations

NCR: Non-coding region
PCG: Protein-coding gene
