# Supplementary figures and images for: Mitochondrial genomes of two Polydora (Spionidae) species provide further evidence that mitochondrial architecture in the Sedentaria (Annelida) is not conserved
Source: Sci Rep. 2021 Jun 30;11:13552. doi: 10.1038/s41598-021-92994-3 (PMC8245539; doi:10.1038/s41598-021-92994-3)

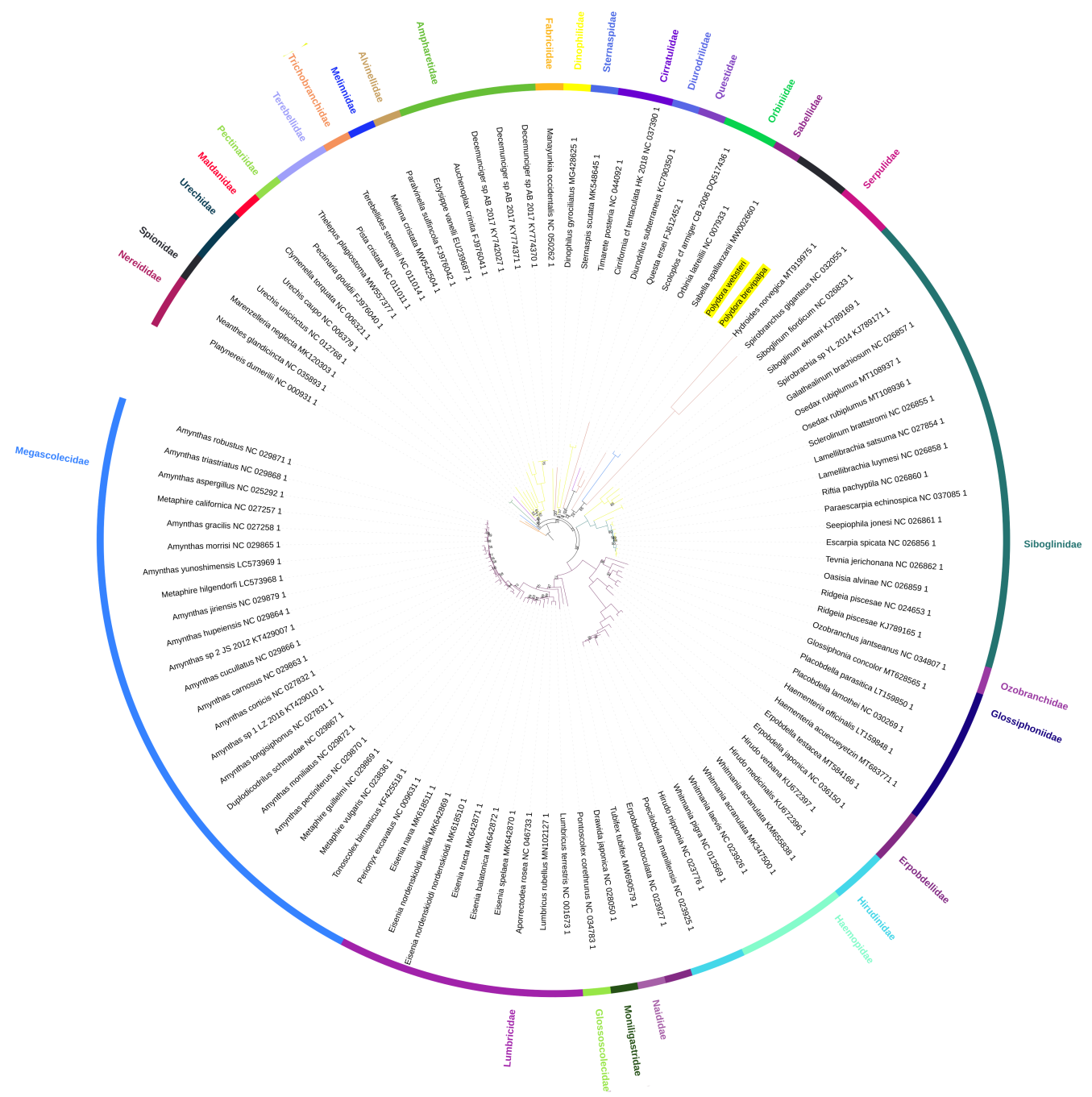

Tree scale: 10

Supplement: Supplementary file 1 — Supplementary Information 1. [file 41598_2021_92994_MOESM1_ESM.pdf]
