# Supplementary material for: Mitochondrial genomes of two Polydora (Spionidae) species provide further evidence that mitochondrial architecture in the Sedentaria (Annelida) is not conserved
Source: Sci Rep. 2021 Jun 30;11:13552. doi: 10.1038/s41598-021-92994-3 (PMC8245539; doi:10.1038/s41598-021-92994-3)
Supplement: Supplementary file 3 — Supplementary Information 3. [file 41598_2021_92994_MOESM3_ESM.pdf]

Supplementary file S3. Rearrangement mechanism and primers.

Mitochondrial genomes of two *Polydora* (Spionidae) species provide further evidence that mitochondrial architecture in the Sedentaria (Annelida) is not conserved

Lingtong Ye, Tuo Yao, Jie Lu, Jingzhe Jiang, Changming Bai

Corresponding author: Dr. Lingtong Ye; E-mail: lingtong2753@126.com

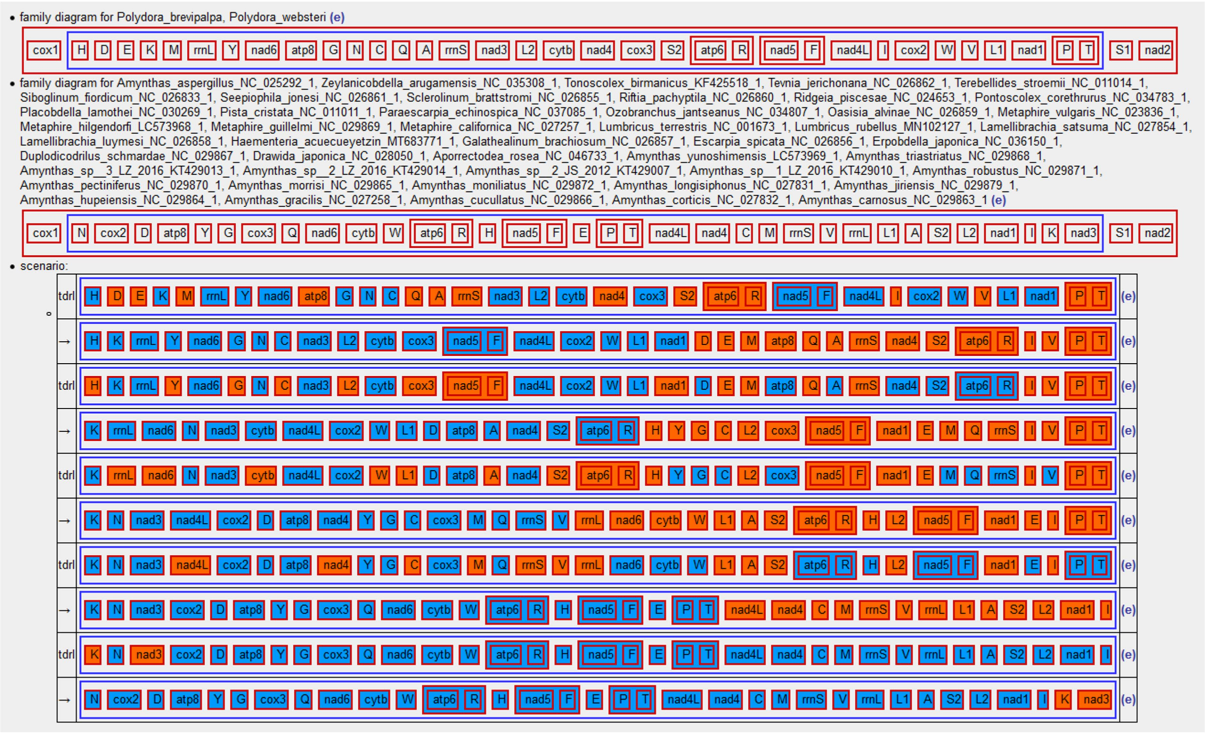

Figure S1. CREx-inferred (breakpoint distance method) scenario of the evolution from the common sedentarian GO to the one observed in the two *Polydora* species.

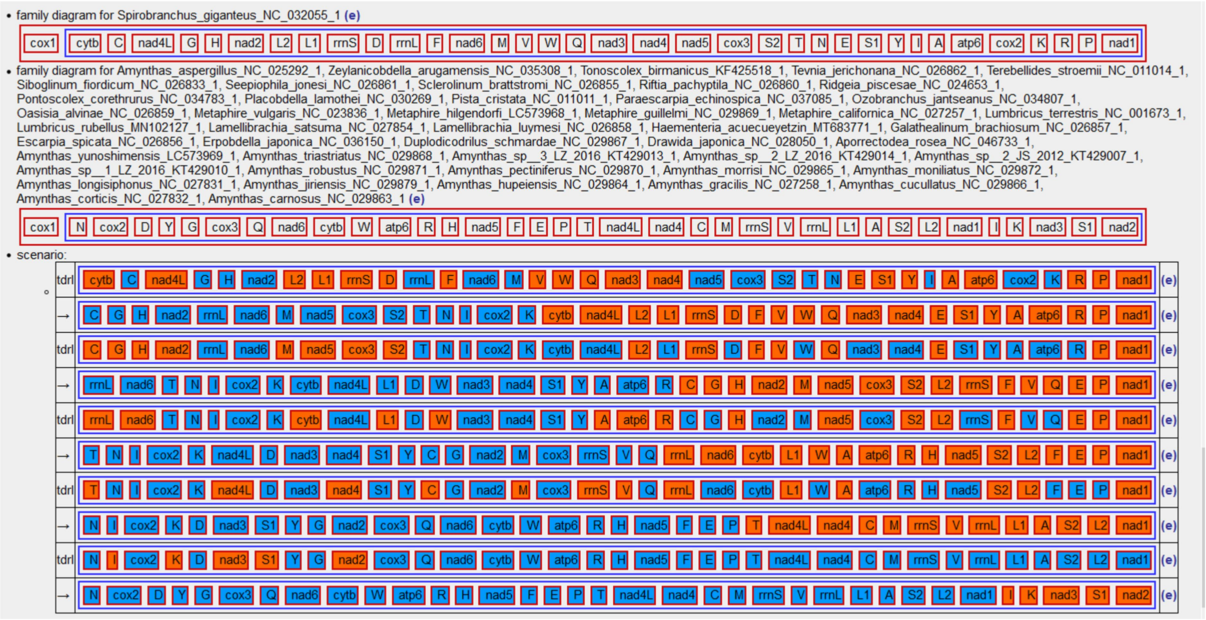

Figure S2. CREx-inferred (breakpoint distance method) scenario of the evolution from the common sedentarian GO to the one observed in *S. giganteus*.

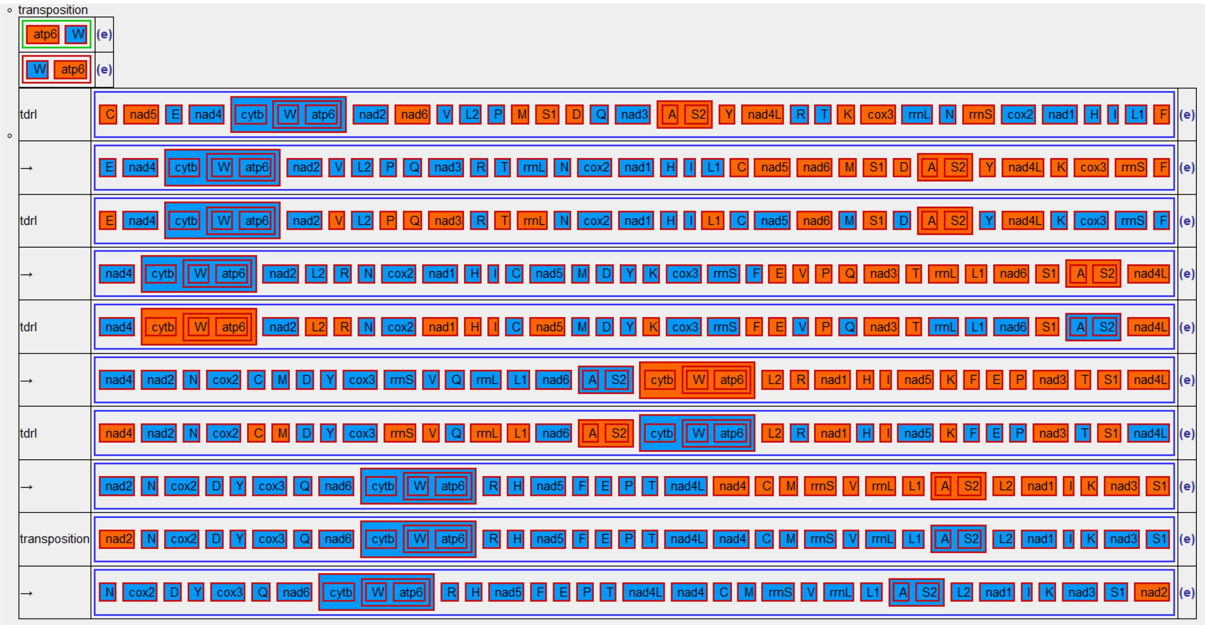

Figure S3. CREx-inferred (breakpoint distance method) scenario of the evolution from the common sedentarian GO to the one observed in *Hydroides norvegica*.

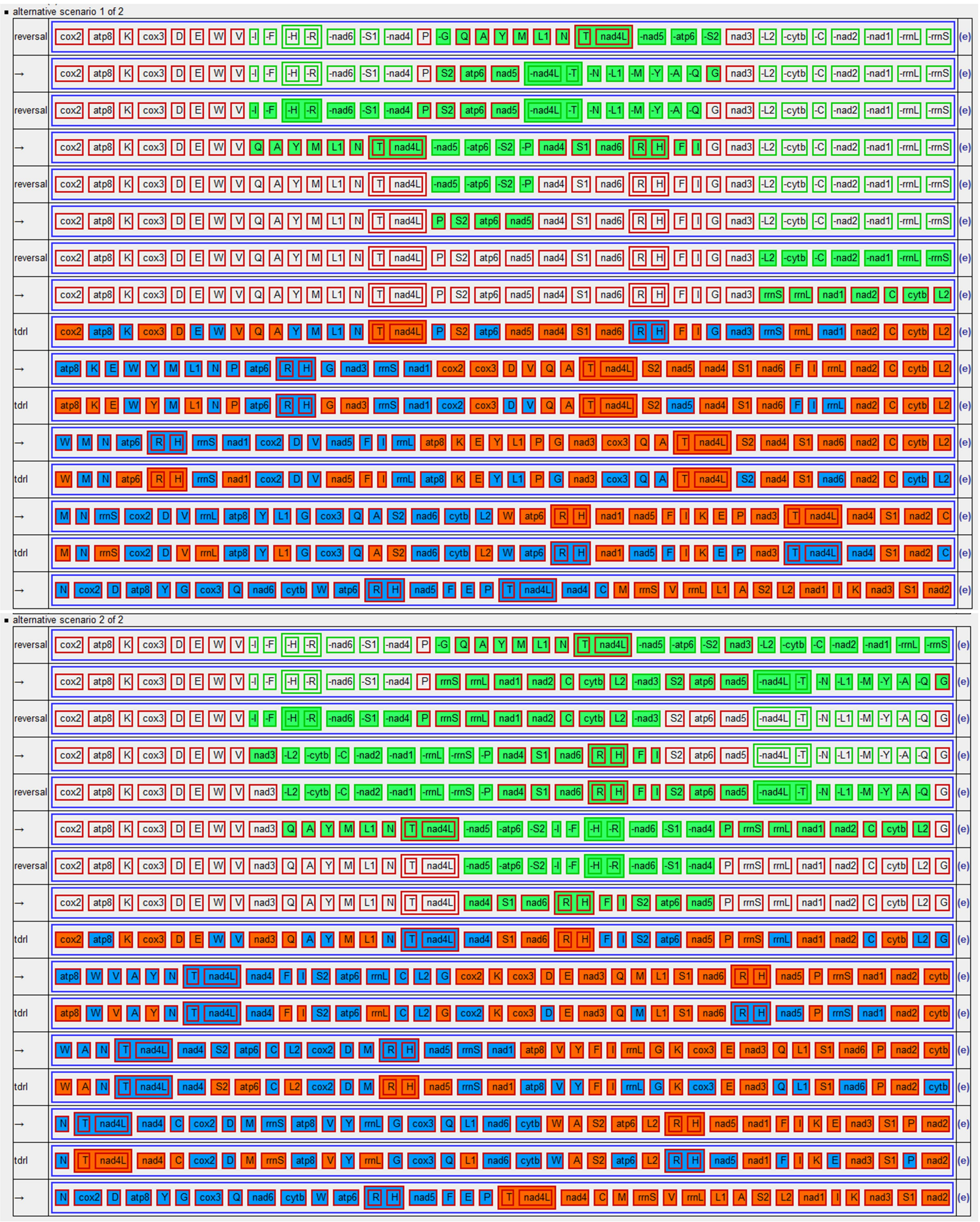

Figure S4. CREx-inferred (breakpoint distance method) scenarios of the evolution from the common sedentarian GO to the one observed in *Sabella spallanzanii*.

Table S1. Primers used for amplification of the mitochondrial genome of *Polydora brevipalpa.*

Fragment No.	Gene/region	Primer name	Sequence (5'-3')	Length (bp)
F1	16S	D3F1	TGWCCGTGCTAAGGTAGCAT	435
		D3R1	GACTTACGWCBGWCRRAACTC	
F2	16S-12S	D3F2	CGATGTTGGCTCAAGGTTAC	2661
		D3R2	GGTTTCACGGGTTTATAGAAGTG	
F3	12S	D3F3	GTGCCAGCTGCCGCGGTCAGAC	582
		D3R3	CCTACCTTGTTACGACTT	
F4	12S-ND4	D3F4	CATTTCTTAATAGTAGTGAAC	2979
		D3R4	GTGAAGAGGGTACTGGAGGAC	
F5	ND4	D3F5	CAACCAGAGCGACTTCAGGC	244
		D3R5	CTACATGGGCTTTAGGGAGCC	
F6	ND4-COX3	D3F6	GTCCTCCAGTACCCTCTTCAC	1235
		D3R6	GCCCTATGAAGAAAGGCTCAG	
F7	COX3	D3F7	AAACTGATGACGYGATAT	458
		D3R7	CATGAAKTCCRTGGAAKCCWG	
F8	COX3-COX2	D3F8	CTACAAAAAGCTACAAGAGC	4322
		D3R8	GACTTCTAATAGGTGTGTTAC	
F9	COX2	D3F9	AAGGWCATCARTGATAYTGA	331

		D3R9	CCACAAATYTCRGARCATTGACC	
F10	COX2-ND1	D3F10	CCTTTACTATCCCTTCTCTAG	748
		D3R10	AGGTTTGCTAGAGCTAGTGC	
F11	ND1	D3F11	CCATTGCTGACGCCATGAAAC	466
		D3R11	GATTCCCCTTCAACTAGATC	
F12	ND1-COX1	D3F12	CGCTTCTTATTCAGAACATTC	1909
		D3R12	CCATCTTCTACTCATATACC	
F13	COX1	D3F13	ATTGGGGGCTTTGGTAACTG	896
		D3R13	ACATAGTATGTGTCGTGGAG	
F14	COX1-16S	D3F14	AGGTGAATAGCAACTGCAAG	2336
		D3R14	CTCTATAGGGTCTTTTCGTC	

Table S2. Primers used for amplification of the mitochondrial genome of *Polydora websteri.*

Fragment No.	Gene or region	Primer name	Sequence (5'-3')	Length (bp)
F1	16S	YJ2F1	TGWCCGTGCTAAGGTAGCAT	429
		YJ2R1	GACTTACGWCBGWCRRAACTC	
F2	16S-12S	YJ2F2	GATGTTGGCTCAAGGTTACAG	2682
		YJ2R2	GTTTATGTTCTAGGTTTCACG	
F3	12S	YJ2F3	GTGCCAGCTGCCGCGGTCAGAC	582
		YJ2R3	CCTACCTTGTTACGACTT	
F4	12S-CYTB	YJ2F4	CTTAATAGTAGTGGAATTC	1923
		YJ2R4	ACGGTACTATATCAGATCTAG	
F5	CYTB	YJ2F5	GATCTGGGGAGGTTTTGCTG	539
		YJ2R5	CTGGGCGTCCGCCAATTCAAG	
F6	CYTB-COX3	YJ2F6	GTACGCCATTCTTCGATCTATCC	2130
		YJ2R6	ATTGGAGGTCACTGACAGGCA	
F7	COX3	YJ2F7	AAACTGATGACGYGATAT	458
		YJ2R7	CATGAAKTCCRTGGAAKCCWG	
F8	COX3-COX2	YJ2F8	GGAAAACTAAGAAAAGCAAC	4336
		YJ2R8	GCTCGGTTAGTAACTTCTAG	

F9	COX2	YJ2F9	AAGGWCATCARTGATAYTGA	331
		YJ2R9	CCACAAATYTCRGARCATTGACC	
F10	COX2-COX1	YJ2F10	CGATGTTCTTCATGCCTTCAC	2913
		YJ2R10	GTTCATCCTGTTCCTGCTC	
F11	COX1	YJ2F11	TTGGAAATTGGTTAATTCC	913
		YJ2R11	CTTAGAACGTAATGGAAGTG	
F12	COX1-16S	YJ2F12	CTGCTAGAGGAACACGACTC	2327
		YJ2R12	CTCTATAGGGTCTTTTCGTC	
